# Histone H3 cysteine 110 enhances iron metabolism and modulates replicative life span in *Saccharomyces cerevisiae*

**DOI:** 10.1126/sciadv.adv4082

**Published:** 2025-04-11

**Authors:** Chen Cheng, Brenna S. McCauley, Nedas Matulionis, Maria Vogelauer, Dimitrios Camacho, Heather R. Christofk, Weiwei Dang, Nicholas A. T. Irwin, Siavash K. Kurdistani

**Affiliations:** ^1^ Department of Biological Chemistry, David Geffen School of Medicine, University of California, Los Angeles, Los Angeles, CA 90095, USA.; ^2^ Huffington Center on Aging, Department of Molecular and Human Genetics, Baylor College of Medicine, Houston, TX 77030, USA.; ^3^ Department of Molecular and Cell Biology, University of California, Berkeley, Berkeley, CA 94720, USA.; ^4^ UCLA Jonsson Comprehensive Cancer Center, Los Angeles, CA 90095, USA.; ^5^ Eli and Edythe Broad Center of Regenerative Medicine and Stem Cell Research, David Geffen School of Medicine, University of California, Los Angeles, Los Angeles, CA 90095, USA.; ^6^ Gregor Mendel Institute (GMI), Austrian Academy of Sciences, Vienna BioCenter (VBC), Vienna, Austria.; ^7^ Department of Pathology and Laboratory Medicine, David Geffen School of Medicine, University of California, Los Angeles, Los Angeles, CA 90095, USA.

## Abstract

The discovery of histone H3 copper reductase activity provides a novel metabolic framework for understanding the functions of core histone residues, which, unlike N-terminal residues, have remained largely unexplored. We previously demonstrated that histone H3 cysteine 110 (H3C110) contributes to cupric (Cu^2+^) ion binding and its reduction to the cuprous (Cu^1+^) form. However, this residue is absent in *Saccharomyces cerevisiae*, raising questions about its evolutionary and functional significance. Here, we report that H3C110 has been lost in many fungal lineages despite near-universal conservation across eukaryotes. Introduction of H3C110 into *S. cerevisiae* increased intracellular Cu^1+^ levels and ameliorated the iron homeostasis defects caused by inactivation of the Cup1 metallothionein or glutathione depletion. Enhanced histone copper reductase activity also extended replicative life span under oxidative growth conditions but reduced it under fermentative conditions. Our findings suggest that a trade-off between histone copper reductase activity, iron metabolism, and life span may underlie the loss or retention of H3C110 across eukaryotes.

## Teaser

Histone H3 cysteine 110 enhances iron metabolism and modulates replicative life span in yeast.

## INTRODUCTION

Histones compact eukaryotic DNA and affect many DNA-dependent processes by controlling access to regulatory DNA sequences and through posttranslational modifications on various residues, particularly within their N-terminal tails (*1*, *2*). Epigenetic machinery, including writers, readers, and eraser proteins, links the presence of these modifications to gene expression and virtually all other DNA-dependent functions (*1*). Although the N-terminal residues and their modifications have been extensively studied, the roles of residues in the core, globular domains of histones are much less understood. A key challenge in studying the core residues has been the lack of clear hypotheses for their functions, especially when they cannot be posttranslationally modified or are distant from modified residues. As a result, studies of core residues often default to the conventional epigenetic framework as it is the only available model for interpreting their roles in chromatin structure and function (*2*).

Previously, we found that histone H3, in complex with histone H4 or within nucleosomes, exhibits catalytic activity as a copper reductase enzyme, facilitating the conversion of cupric (Cu^2+^) to cuprous (Cu^1+^) ions (*3*, *4*). This activity is important for effective distribution of copper to various copper-dependent enzymes in the cytoplasm and mitochondria as intracellular copper chaperones specifically bind the reduced, Cu^1+^ form of copper ions (*5*). This finding has introduced a novel metabolic framework for exploring chromatin structure and function. Through this new enzymatic perspective, we recently uncovered the role of an otherwise unremarkable residue, histone H3 leucine 126 (H3L126), which acts as the axial ligand for copper binding and is essential for fine-tuning the nucleosome enzyme activity (*3*). Recognizing this residue and discerning its function would have been challenging within the conventional epigenetic framework.

Histone H3 cysteine 110 (H3C110) is a unique core residue, being the only cysteine present in the four major core histones (H2A, H2B, H3, and H4), except for H3C96, which is found in the histone H3.1 variant in mammals. H3C110 is located at the interface of two opposing histone H3 proteins within the nucleosome, positioned 6.2 Å from the adjacent cysteine. This distance prevents it from contributing to the H3-H3′ interaction and makes the formation of a disulfide bridge unlikely (*6*). A nucleosome consists of a tetramer of histones H3 and H4, along with two pairs of H2A-H2B dimers, collectively wrapping 146 base pairs of DNA. The histone H3-H4 tetramer is itself composed of two dimers of H3 and H4 histones that interact exclusively through histone H3 residues. We identified the H3-H3′ interface, where the H3-H4 dimers interact, as the probable enzyme active site as it is where Cu^2+^ binds and is likely reduced (*4*). H3C110 is crucial for optimal copper binding, and mutating this residue to alanine substantially reduced the enzymatic activity of human H3-H4 tetramers in vitro (*4*).

The histone H3 of *Saccharomyces cerevisiae* lacks this cysteine, instead having an alanine at the equivalent position (H3A110). However, genetic, molecular, and biochemical data indicated that yeast histone H3 still functions as a copper reductase in cells (*3*, *4*, *7*). Introducing the cysteine through the H3A110C mutation significantly increased the enzyme activity of yeast histone H3 in vitro and acted as a gain-of-function mutation in vivo (*4*). These findings raised important questions about why budding yeast lacks this cysteine and the broader evolutionary pattern of its conservation.

Moreover, in eukaryotes, copper homeostasis does not occur in isolation as the regulation of other metals such as iron is interdependent with copper (*8*). Several genes involved in copper homeostasis also exhibit transcriptional responses to changes in iron levels (*9*). Copper is essential for iron uptake through the activity of the copper-dependent Fet3 iron oxidase in yeast (*10*) and plays a role in intracellular iron mobilization, although the precise mechanisms remain unclear. This, in turn, raises additional questions about the role of H3C110 in a broader metabolic context and leads to the hypothesis that iron homeostasis and intracellular redox regulation may also be affected by H3C110.

To investigate the evolutionary history and cellular consequences of H3C110, we conducted a phylogenetic analysis of H3C110 and studied the biological effects of reintroducing this residue into budding yeast. We found that H3C110 has been nearly universally conserved throughout eukaryotic evolution but has notably been lost in most fungi, including the Saccharomycetaceae lineage. However, some eukaryotes have histone H3 variants with and without H3C110, suggesting that histone variants may enable the functional regulation of H3C110 activity. The loss of H3C110 correlates with the absence of a similarly positioned cysteine in the centromeric histone H3 variant (cenH3C204). The reintroduction of C110 into yeast histone H3 (i.e., H3A110C mutation) led to increased intracellular levels of Cu^1+^ in cells where the copper-binding protein, the Cup1 metallothionine, was inactivated to prevent adsorption of cuprous ions. Unexpectedly, the expression of iron regulon genes was increased upon Cup1 inactivation, signifying an iron deficiency response. However, the H3A110C mutation mitigated this response, significantly improving the growth of Cup1 mutant strains, especially in oxidative media when demand for iron is high. This effect of H3C110 was generalizable as the H3A110C mutation also alleviated the iron deficiency response resulting from glutathione deficiency. The H3A110C mutation also improved the growth of strains deficient in copper delivery to cytochrome c oxidase, linking the nucleosome copper reductase directly to mitochondria. We further found that the H3A110C mutation extended the replicative life span (RLS) of yeast under oxidative growth conditions but reduced it in fermentative media. Another copper reductase gain-of-function mutation, H3L126M, exhibited similar phenotypes of improving iron homeostasis and modulating life span. Our results suggest that increasing the copper reductase activity of nucleosomes enhances iron homeostasis, benefiting cells in oxidative environments while potentially reducing fitness under fermentative conditions.

## RESULTS

### The histone H3C110 residue is lost in most fungi

**Fig. 1.**
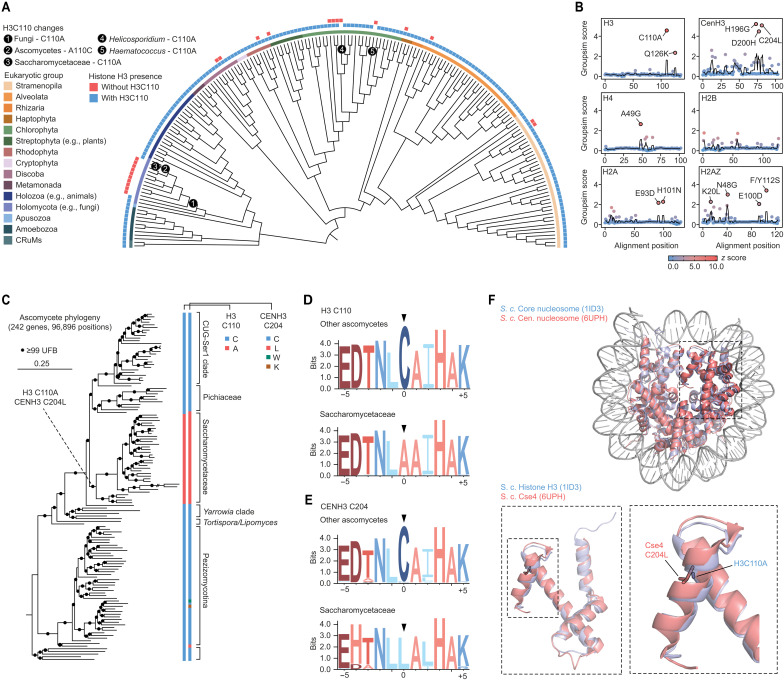
Histone H3C110 is absent in many fungi. (**A**) Distribution of core histone H3 across eukaryotes, highlighting the presence of paralogs without (red squares, outer circle) or with (blue squares) H3C110. Different eukaryotic groups are color coded as indicated, and H3C110 divergence events are noted. (**B**) Identification of histones residues that distinguish the Saccharomycetaceae, which lack H3C110, from other ascomycetes. The groupsim score quantifies how well a residue defines these two groups, and the *z*-score coloring compares each residue’s score to the average across all residues within a given histone. Residues with scores above 2.0 and *z* scores above 5.0 are noted. (**C**) Distribution of H3C110 and centromeric (CEN) histone H3C204 across ascomycetes. The phylogeny was inferred based on a concatenated matrix of 242 proteins using the Q.yeast+I+R10 substitution model. Statistical support was inferred using ultrafast bootstraps (*n* = 1000), and the scale bar represents the average number of substitutions per site. (**D** and **E**) Sequence logos depicting the conservation of five residues upstream and downstream of H3C110 and CenH3C204 in ascomycetes compared to the Saccharomycetaceae. (**F**) Crystal structures of core [Protein Data Bank (PDB): 1ID3] and centromeric (PDB: 6UPH) nucleosomes from *S. cerevisiae*, demonstrating the similar positioning of H3C110 (H3A110) and CenH3C204 (CenH3L204) within the nucleosome. Full phylogenies and species names that can be visualized are available from iTOL: https://itol.embl.de/shared/eLOrhXQmeWM3.

To determine the distribution of H3C110 across eukaryotes, we assessed histone sequences derived from species representing the breadth of eukaryotic diversity (Fig. 1A). H3C110 encoding core histones were identified across most eukaryotes, with the exception of multiple fungal lineages and a few green algal species including *Haematococcus* and *Helicosporidium*. Nearly all fungi lacked H3C110, except for the Cryptomycota, represented by *Rozella*, and some members of the Ascomycota. A more detailed examination of the H3C110 distribution across the ascomycetes revealed that, although lineages retain H3C110, it diverged in the Saccharomycetaceae, a group including *S. cerevisiae*. Together, this distribution can most parsimoniously be explained by an H3C110 substitution in a fungal ancestor following the divergence of the Cryptomycota, followed by an H3C110 reversion in the ascomycetes, and a subsequent substitution in the Saccharomycetaceae. In addition, some eukaryotes, including several algal lineages such as *Nannochloropsis* and *Coccomyxa*, as well as the trypanosomatids *Strigomonas* and *Angomonas*, have histone H3 variants with and without H3C110 (Fig. 1A). These paralogs could provide a potential regulatory mechanism for controlling the presence and, in turn, activity of H3C110.

Given the multimeric nature of the nucleosome, and the importance of histone residues for inter-histone contact, particularly within H3-H3′ dimerization region (*6*, *11*), we sought to identify additional residues whose diversification correlates with that of H3C110. By comparing the amino acids of each core histones and histone variants CenH3 (Cse4) and H2AZ, in ascomycetes with and without H3C110 using the groupsim metric, we identified several residues that diverged at a similar point in time as H3C110 (Fig. 1B). However, only a single residue in CenH3, CenH3C204, was inferred to have diverged at approximately the same time as H3C110 into a leucine (CenH3C204L) (Fig. 1, C to E). In contrast, additional residues with high groupsim scores were only correlated with H3C110 divergence but were substituted at alternative points in time, such as CenH3D200H (Fig. 1E). Notably, in *S. cerevisiae*, H3A110 and CenH3L204 occupy equivalent positions near the C terminus of histone H3 (Fig. 1F), implying that similar evolutionary pressures may have driven convergent functional changes in both H3 paralogs.

### H3C110 increases intracellular Cu^1+^ levels

To investigate the biological effects of H3C110, we introduced the H3A110C mutation in chromosomal copies of the two histone H3 genes in yeast, HHT1 and HHT2, leaving the genes under their natural promoters, generating the *H3^A110C^* strain. Gene expression analysis in fermentative [synthetic complete (SC)] or oxidative medium [SC with ethanol and glycerol (SCEG)] revealed only one gene, *MIC19*, that is significantly up-regulated in the *H3^A110C^* strain compared to wild-type (WT) (fig. S1, A and B). *MIC19* encodes a component of the MICOS complex, a mitochondrial inner membrane complex that helps maintain crista junctions (*12*). Deleting *MIC19* (*mic19*Δ) in *H3^A110C^* did not preferentially affect viability or growth under oxidative conditions compared to *mic19*Δ alone (fig. S1C). The significance of *MIC19* up-regulation in *H3^A110C^* remains unclear.

**Fig. 2.**
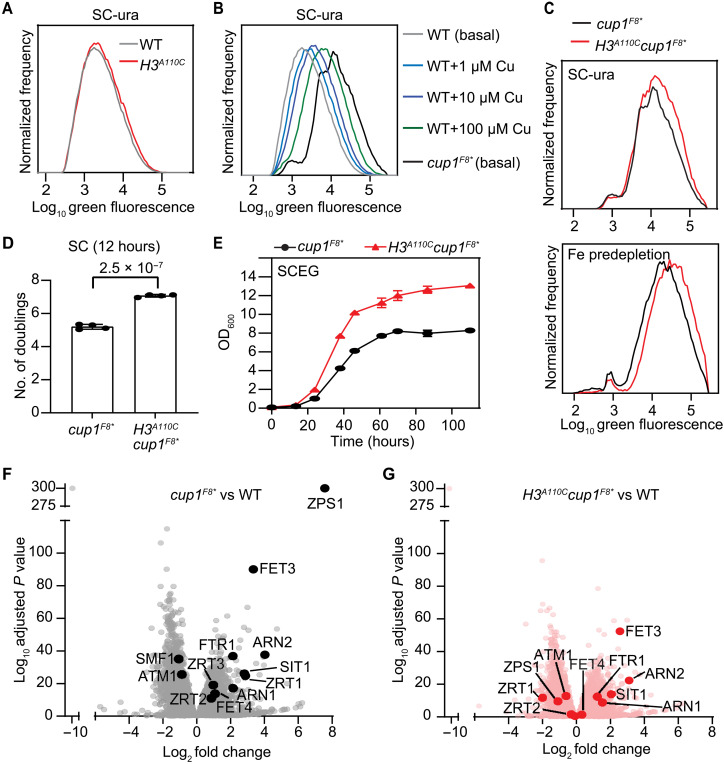
Introduction of H3C110 into *S. cerevisiae* increases intracellular Cu^1+^ levels. (**A** to **C**) Average flow cytometry distributions of cells containing the p(CUP1)-GFP plasmid grown in the indicated media from eight experiments. Baseline copper and iron concentrations in SC are ~0.2 and 1.2 μM, respectively. (**D**) Bar graph depicts the number of doublings in fermentative medium (SC) for the indicated strains from four experiments. *P* value was calculated using a *t* test. (**E**) Growth curves of the indicated strains in oxidative medium (SCEG). Baseline copper level in SCEG is 0.25 μM. OD_600_, optical density at 600 nm. (**F** and **G**) Volcano scatterplots illustrating the relationship between change in gene expression of the indicated strains and the corresponding level of significance. Several genes involved in iron or zinc homeostasis are indicated.

Next, to assess whether the H3A110C mutation affected intracellular Cu^1+^ abundance, we used a previously constructed reporter plasmid (*4*) (fig. S2A). This plasmid contains a green fluorescent protein (GFP) gene under the control of the *CUP1* promoter, a main target of Cup2, a transcription factor that is directly activated by Cu^1+^ but not Cu^2+^ ions (*13*, *14*). In both fermentative (SC) and oxidative (SCEG) media, GFP expression was similar between the WT and *H3^A110C^* strains (Fig. 2A and fig. S2B). This led us to consider whether the effects of H3A110C on Cu^1+^ levels were being masked by sequestration of Cu^1+^ ions by the Cup1 metallothionein (*15*, *16*). To test this, we introduced a stop codon into every copy of *CUP1* (cup1^F8*^) to eliminate Cup1 function (*7*). This resulted in a substantial increase in GFP expression, equivalent to the addition of over 100 μM exogenous copper, likely highlighting Cup1’s significant capacity to buffer intracellular Cu^1+^ levels (Fig. 2B). In the cup1^F8*^ background, the H3A110C mutation showed even higher GFP levels, an effect that was further enhanced by predepleting the media of iron (Fig. 2C). These findings indicate that introduction of H3C110 increases intracellular Cu^1+^ levels.

Considering that intracellular Cu^1+^ can be toxic at high levels (*17*, *18*), we expected the H3A110C mutation to weaken the cup1^F8*^ strain due its already elevated Cu^1+^ ions. However, contrary to our expectation, the H3A110C mutation significantly improved the growth of cup1^F8*^ in both fermentative (Fig. 2D) and oxidative media (Fig. 2E), where the demand for iron and copper is higher due to increased mitochondrial respiration. Addition of a small amount of exogenous copper also enhanced the growth of the cup1^F8*^ strains, with cells reaching higher densities (fig. S2C).

To understand the molecular basis of this effect, we performed gene expression and ontology analyses, which revealed significant enrichment of iron homeostasis genes among the up-regulated genes in cup1^F8*^, indicating an activation of iron deficiency response (Fig. 2F and fig. S2, D and E). In yeast, the iron regulon is activated in response to a decrease in the production or supply of iron-sulfur (Fe-S) clusters (*19*)—molecular assemblies of iron and sulfur produced in mitochondria that serve as enzymatic and structural cofactors in numerous proteins throughout the cell (*20*). Several genes that are typically induced in response to zinc deficiency (*21*, *22*) are also up-regulated in the cup1^F8*^ strain compared to WT (Fig. 2F and fig. S2, D and E). The up-regulation of the iron and zinc regulons indicates a previously unrecognized role for Cup1 in iron and zinc homeostasis. In the cup1^F8*^ background, the H3A110C mutation lowered the expression of iron and zinc regulon genes, indicating that introduction of H3C110 improved iron and zinc homeostasis (Fig. 2G and fig. S2F), likely explaining the improved growth of the cup1^F8*^ strain with the H3A110C mutation.

### H3C110 improves iron homeostasis and alleviates metabolic stress during glutathione deficiency

**Fig. 3.**
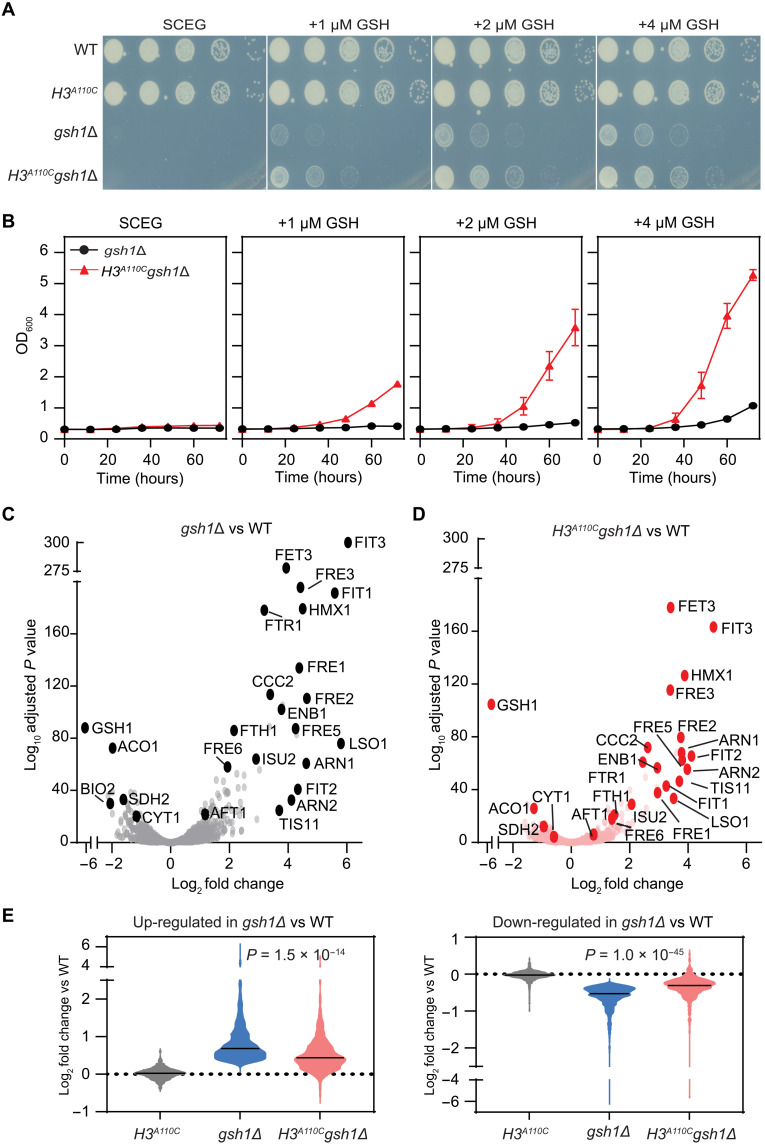
Introduction of H3C110 into *S. cerevisiae* decreases glutathione requirement. (**A**) Spot test assays of the indicated strains in oxidative medium (SCEG) or with glutathione (GSH) supplementation as indicated. Note that SCEG contains no glutathione. (**B**) Growth curves of the indicated strains in SCEG or with GSH supplementation as indicated. (**C** and **D**) Volcano scatterplots illustrating the relationship between changes in gene expression of the indicated strains and the corresponding level of significance. Several genes involved in iron homeostasis are indicated. (**E**) Violin plots showing the average mRNA expression of genes significantly (*P* < 0.05) up-regulated (left) or down-regulated (right) in *gsh1*Δ versus WT in the indicated strains. The solid line indicates the median value. *P* values were calculated using a *t* test.

Glutathione plays a key role in supporting Fe-S cluster biogenesis by facilitating iron uptake into mitochondria (*23*). To determine whether the H3A110C mutation could improve iron homeostasis in the absence of adequate glutathione, we deleted the γ-glutamylcysteine synthetase (*GSH1*) gene, which catalyzes the first step in glutathione biosynthesis (*24*), in both WT and *H3^A110C^* strains, and examined their growth on solid and in liquid oxidative media. Neither the *gsh1*Δ nor the *H3^A110C^gsh1*Δ strain was able to grow in medium without supplemental glutathione. However, adding low levels of glutathione rescued their growth defect, with the *H3^A110C^gsh1*Δ strain requiring significantly less glutathione to restore respiratory growth compared to the *gsh1*Δ strain (Fig. 3A). This effect was consistent across both solid and liquid media (Fig. 3B), as well as in an independently derived *H3^A110C^gsh1*Δ strain in a different strain background (fig. S3A). In addition, the H3L126M mutation, which also enhances the copper reductase activity of nucleosomes (*3*), similarly reduced the requirement for glutathione supplementation in the *gsh1*Δ strain (fig. S3B). Together, these results suggest that enhancing histone H3 copper reductase activity lowers the need for glutathione supplementation in yeast strains lacking the ability to synthesize it.

To determine whether the effect of H3C110 on lowering glutathione requirement of *gsh1*Δ strain was related to the activity of glutathione reductase, we deleted the *GLR1* gene, which encodes glutathione reductase 1 in WT and *H3^A110C^* strains. Unlike the *gsh1*Δ strain, *glr1*Δ cells did not exhibit a growth defect in fermentative or oxidative media lacking glutathione, making it difficult to assess the impact of the H3A110C mutation (fig. S3C). To overcome this, we generated a *glr1Δgsh1*Δ double mutant, which required significantly more supplemental glutathione for growth under oxidative conditions (fig. S3D). Despite this increased dependency, the H3A110C mutation (*glr1Δgsh1ΔH3^A110C^*) still reduced the glutathione requirement, indicating that the effects of H3C110 are independent of Glr1 activity.

Gene expression and ontology analyses revealed a significant up-regulation of the iron regulon in the *gsh1*Δ strain (Fig. 3C and fig. S3, E and F), consistent with the expected impairment of Fe-S cluster metabolism. Consistent with its effects in the cup1^F8*^ background, the H3A110C mutation reduced the expression of iron regulon genes in the *gsh1*Δ strain (Fig. 3D and fig. S3G), indicating an improvement in iron homeostasis. A broader analysis showed that the H3A110C mutation partially restored the gene expression changes observed in *gsh1*Δ back toward WT levels. Genes that were up-regulated in *gsh1*Δ returned partially to normal levels, whereas down-regulated genes also showed partial restoration in the presence of the H3A110C mutation (Fig. 3E). These findings suggest that the reduced need for supplemental glutathione in the *H3^A110C^gsh1*Δ strain is associated with an overall shift in gene expression toward a WT pattern including that of the iron regulon.

### Rescue of *gsh1*Δ by H3A110C is not linked to iron uptake but to improvements in cellular redox balance

**Fig. 4.**
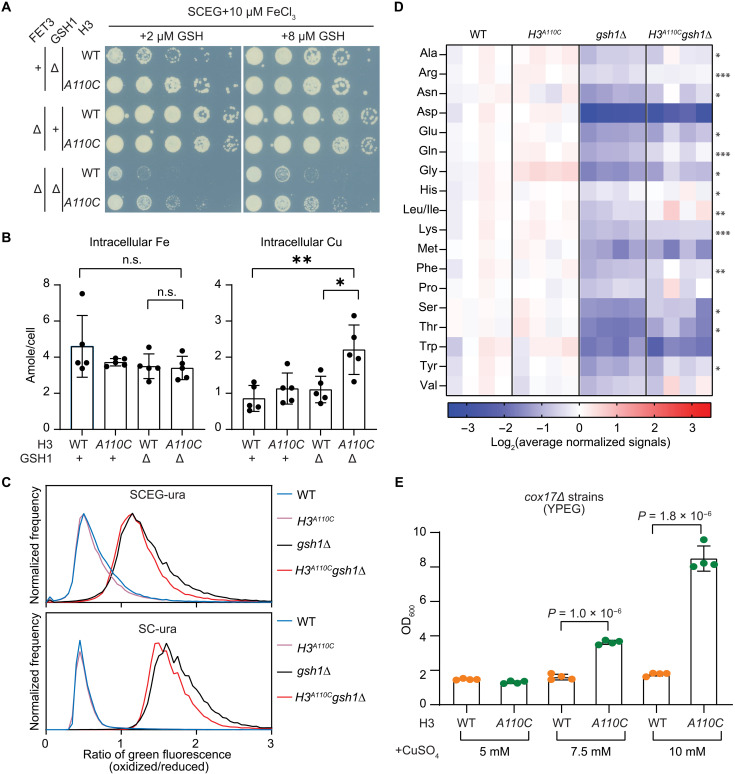
Introduction of H3C110 into *S. cerevisiae* improves cellular redox and metabolic profile under glutathione deficiency. (**A**) Spot test assays of the indicated strains in oxidative medium (SCEG) with iron supplementation. Note that the baseline levels of iron in SCEG do not support the growth of *fet3*Δ strains. (**B**) Intracellular iron and copper content of exponentially growing strains as measured by ICP-MS/MS. Bars are means ± SD from five independent experiments (*P < 0.05; **P < 0.01, *t* test). n.s., not significant. (**C**) Average normalized flow cytometry distributions of cells containing the redox-sensitive ro2GFP grown in the indicated media from eight experiments. The *x* axis indicates the ratio of oxidized/reduced green fluorescence signal. (**D**) Heatmap showing intracellular levels of the indicated amino acids in the specified strains from four independent experiments. The scale represents log_2_ average-normalized levels of each amino acid. *P* values indicate the significance of comparisons between *gsh1*Δ and *H3^A110C^gsh1*Δ data points (*P < 0.05, **P < 0.01, ***P < 0.005, *t* test). (**E**) Bar graph depicts the OD_600_ for the indicated strains and media after 5 days of growth, based on four cultures. *P* values were calculated using a *t* test.

To explore how the H3A110C mutation enhances iron homeostasis, we first investigated whether Fet3, a copper-dependent iron oxidase required for iron uptake (*10*), was involved. Although *fet3*Δ did impair the growth of both *gsh1*Δ and *H3^A110C^gsh1*Δ, due to further limitations in iron availability, the H3A110C mutation still reduced the need for glutathione supplementation in the *fet3Δgsh1*Δ strain, ruling out a role for Fet3-mediated iron uptake (Fig. 4A). In addition, we observed no changes in total intracellular iron levels as measured by inductively coupled plasma mass spectrometry (ICP-MS) (*25*), confirming that the H3A110C mutation does not affect iron uptake (Fig. 4B). The *H3^A110C^gsh1*Δ strain exhibited higher levels of intracellular copper, although the nature and intracellular localization of this copper remain to be determined (Fig. 4B).

We next investigated whether the H3A110C mutation enhances glutathione import into mitochondria. This process is mediated by the transporter SLC28A39, which depends on its ability to sense or bind Fe-S clusters through four cysteine residues in its matrix-facing region (*26*). To test this, we mutated two of these cysteines in the orthologous gene, *MTM1* (*27*), in both *gsh1*Δ and *H3^A110C^gsh1*Δ strains. Despite the mutations in *MTM1*, the H3A110C mutation still improved the growth of *gsh1*Δ in this background (fig. S4A), suggesting that enhanced mitochondrial glutathione uptake is unlikely to be the mechanism behind the improved iron homeostasis in the *H3^A110C^gsh1*Δ strain.

Given glutathione’s role in regulating cellular redox balance and the interplay of redox balance with copper and iron homeostasis (*23*), we used a redox-sensitive GFP to assess the cellular oxidation state (*28*). In oxidative, but not fermentative medium, *H3^A110C^* showed a small but reproducible decrease in GFP oxidation compared to WT cells (Fig. 4C and fig. S4B). As expected, *gsh1*Δ cells displayed a substantial increase in oxidized GFP signal in both media, reflecting glutathione depletion (Fig. 4C). In *H3^A110C^gsh1*Δ cells, there was a modest shift toward lower GFP oxidation, indicating that the H3A110C mutation reduces intracellular oxidation in *gsh1*Δ cells (Fig. 4C). A similar effect was observed in the cup1^F8*^ strain (fig. S4C). Although cup1^F8*^ did not show substantially higher GFP oxidation compared to WT, the H3^A110C^cup1^F8*^ exhibited lower GFP oxidation than cup1^F8*^ in oxidative but not fermentative medium (fig. S4C). This suggests that the effects of H3A110C on cellular oxidation state may be a common phenotype shared by cup1^F8*^ and *gsh1*Δ cells.

We next measured over 200 cellular metabolites to investigate the effects of the H3A110C mutation on cellular metabolism in cells grown in oxidative media (data S1). As expected, levels of glutathione and related metabolites were significantly reduced in both *gsh1*Δ and *H3^A110C^gsh1*Δ cells compared to WT or *H3^A110C^* cells (fig. S4D). Among the major metabolites, we observed a significant depletion of amino acids in *gsh1*Δ cells, with most of these amino acid levels being modestly restored in *H3^A110C^gsh1*Δ cells (Fig. 4D). Many other metabolites showed similar trends (fig. S4E).

Together, our data suggest that the reduced need for supplemental glutathione in *H3^A110C^gsh1*Δ cells is associated with a general alleviation of the impact of glutathione deficiency on gene expression including the iron regulon, cellular redox balance, and metabolite levels such as amino acids.

### Histone H3C110 compensates for defects in mitochondrial copper delivery

Considering that mitochondria are the site of Fe-S cluster synthesis and play a critical role in cellular redox balance, we next asked whether the effects of H3C110 could be linked more directly to mitochondrial function. To test this, we deleted the *COX17* gene, which encodes a copper metallochaperone required for delivery of copper to cytochrome c oxidase (complex IV) in mitochondria. The *cox17*Δ strain is unable to grow on oxidative medium without high levels of exogenous copper in the medium (*29*). However, we found that introducing H3A110C into the *cox17*Δ strain (*cox17Δ H3^A110C^*) substantially improved growth in oxidative medium [yeast peptone ethanol glycerol (YPEG)] compared to *cox17*Δ alone (Fig. 4E). This finding suggests that H3C110 and the copper reductase activity of histone H3 are directly linked to mitochondrial function, specifically complex IV activity.

### Histone H3C110 modulates RLS

**Fig. 5.**
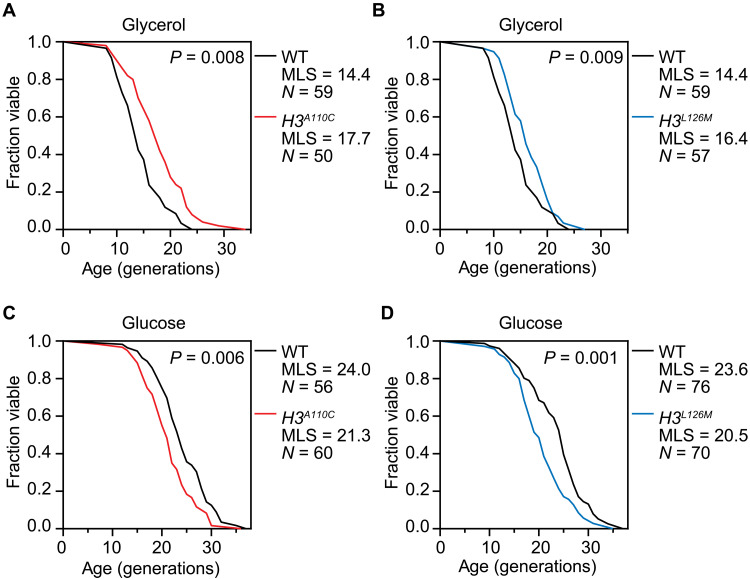
Gain-of-function mutations in the copper reductase activity of histone H3 extend RLS in oxidative medium but reduce it in fermentative medium. (**A** and **B**) RLS analysis of strains with H3A110C or H3L126M mutation grown in oxidative medium (YPGlycerol). Median life span (MLS) in days and the number of cells analyzed are indicated. (**C** and **D**) Same as in (A) and (B), but for cells cultured in fermentative medium (YPD). *P* values were calculated using the Wilcoxon rank sum test.

Yeast RLS, defined as the number of times a mother cell buds to produce progeny daughter cells, has been a valuable model for aging research (*30*). Previous studies have shown that iron homeostasis plays a key role in RLS, with iron supplementation delaying aging and iron limitation accelerating it, primarily through its effects on mitochondrial function (*31*–*33*). Mitochondria are critical for oxidative growth, and consistently, increased iron uptake extends RLS under oxidative, but not fermentative, conditions (*34*). Given that the H3A110C mutation improves iron homeostasis, we used a combination of microfluidic single-cell analysis and high-resolution time-lapse microscopy to measure its impact on yeast RLS (*35*, *36*). In oxidative medium, the H3A110C mutation extended RLS by ~23% compared to WT cells in two independent experiments (Fig. 5A and fig. S5A). Similarly, the H3L126M mutation, which also enhances nucleosome copper reductase activity, extended RLS by about 14% in two separate experiments under the same oxidative growth condition (Fig. 5B and fig. S5B). The opposite effect on RLS was observed when the strains were grown under fermentative conditions. Both H3A110C and H3L126M mutations reduced RLS by ~13% in two independent experiments (Fig. 5, C and D, and fig. S5, C and D). These findings suggest that, although enhancing the copper reductase activity of histone H3 extends RLS during respiratory growth, this benefit comes at the cost of reduced RLS in the presence of glucose, the preferred carbon source.

## DISCUSSION

Histone H3 copper reductase activity offers a novel paradigm for understanding the contributions of histones to the evolution, structure, and functions of chromatin (*37*). By viewing the nucleosome through the lens of histone H3 enzyme activity, we can identify previously unrecognized features and generate novel hypotheses—insights that are not easily derived from the epigenetic framework centered around histone regulation of DNA functions. We have previously demonstrated the utility of this metabolic model by identifying the H3L126 residue as the axial ligand for copper binding, which fine-tunes nucleosome enzymatic activity (*3*). In this study, we applied this metabolic framework to investigate the role of the H3C110 residue, the only cysteine in canonical core histones. The H3.1 mammalian variant histone contains a second cysteine. We found that the presence of H3C110 and the resulting enhancement of histone H3 copper reductase activity contribute to improved iron homeostasis, benefiting growth and replication under oxidative conditions. However, this advantage comes with a trade-off under fermentative conditions, where the same activity may impair RLS. This functional trade-off may explain the evolutionary pattern of H3C110 retention, loss, or regain observed in eukaryotes, especially across the fungal kingdom.

The H3C110 residue is located near the H3-H3′ interface but is not predicted to contribute to the stability of this surface (*6*). In addition, the two opposing cysteines are too far apart to readily form a disulfide bond (*6*). Although disulfide-bonded histone H3 has been observed in nucleosome preparations from cells (*38*), such bonding would require significant displacement of protein-protein and protein-DNA interactions, making it unlikely under normal cellular conditions (*6*). However, despite its position deep within the nucleosomes, H3C110 is accessible as modifications such as S-acylation (*39*), glutathionylation (*40*), S-sulfenylation, and other oxidative modifications (*41*–*43*) have been reported.

The role of H3C110 had remained unknown until we found that it plays a key role in Cu^2+^ binding and its catalysis to Cu^1+^. Although the absence of this cysteine significantly reduced the copper reductase activity of human histone H3 in vitro, it did not completely abolish the activity (*4*). Consistently, genetic and molecular data from the budding yeast, whose histone H3 lacks the C110 residue, supported the notion that it also functions as a copper reductase enzyme (*4*, *7*). This finding raised the question of what evolutionary pressures have driven the loss of this residue in yeast.

We addressed this question by introducing the H3C110 residue into yeast histone H3, replacing the naturally occurring alanine. In a previous work, we demonstrated that the H3A110C mutation increases the copper reductase activity of yeast histone H3 in vitro and acts as a gain-of-function mutation in vivo (*4*). Here, we further characterized the *H3^A110C^* strain and found that it increases intracellular Cu^1+^ levels and improves iron homeostasis. This improvement was evident when intracellular balance was disrupted, such as by inactivation of the Cup1 metallothionein or reduction of the glutathione pool, and was independent of iron uptake. These findings suggest that H3C110 may regulate iron metabolism by influencing intracellular iron distribution or utilization rather than directly affecting iron uptake. In both cup1^F8*^ and *gsh1*Δ cells, the H3A110C mutation also made the overall cellular milieu more reducing. Whether this effect results from improved iron homeostasis or contributes to it—potentially by preventing the oxidation of Fe-S clusters—remains to be determined. It is also possible that the ability of H3C110 to compensate for defects in mitochondrial copper delivery is linked to its role in improving iron homeostasis.

The improvement in intracellular iron homeostasis may explain the extension of RLS under oxidative growth conditions, where the demand for iron is high due to increased mitochondrial activity. However, as an oxidoreductase enzyme, histone H3 consumes reducing equivalents that could otherwise be used by various anabolic pathways (*44*). Thus, the improved iron homeostasis likely comes at the cost of increased consumption of reducing equivalents. Consistent with this, under fermentative conditions—where the demand for iron is lower—the enhanced copper reductase activity of histone H3 shortens RLS. It is conceivable that the trade-off between improved iron metabolism and the consumption of reducing equivalents by histone enzyme activity may represent the molecular cost-benefit balance behind the evolution and function of H3C110 in eukaryotes.

## MATERIALS AND METHODS

### Preparation of solutions and glassware

Removal of contaminating metals from glassware and solutions is a critical precaution to ensure the reproducibility of experiments. All glassware was treated with 3.7% hydrochloric acid for ≥12 hours, followed by 7% nitric acid for ≥12 hours and extensive rinsing with Milli-Q (MilliporeSigma) ultrapure water to remove trace metal contamination. All solutions, buffers, and washes were prepared and done using Milli-Q ultrapure water. Solutions were prepared using BioUltra grade (Sigma-Aldrich) reagents, when available. For yeast media, the addition of all components was done without the use of metal spatulas. Media were filtered through 0.2-μm membranes.

### Strain generations and growth conditions

All *S. cerevisiae* strains used in this study are either based on OCY1131, which was generated from BY4741, or YLK1879 (both are from the S288C background) (*4*). A complete list of strains and their genotypes is listed in table S1. The CRISPR-Cas9 system optimized for *S. cerevisiae* was used to generate the H3A110C and MTM1 C49/51A mutations and to delete *GLR1* (*45*). *GSH1*, *FET3*, *COX17*, and *MIC19* were deleted by a standard gene replacement and targeted insertion methodology using selectable marker integration (*46*, *47*). All strains were routinely maintained on YPD (1% yeast extract, 2% peptone, and 2% d-glucose) plates. Fermentative media were synthetic complete medium (SC) consisting of yeast nitrogen base (BD Biosciences or Sunrise Science), ammonium sulfate (Sigma-Aldrich), 2% glucose (Sigma-Aldrich), all amino acids, uracil, and adenine (Sunrise Science) and SC lacking uracil (SC-ura). For oxidative media, 2% glucose was replaced with 2% each of ethanol and glycerol (SCEG, SCEG-ura, or YPEG). All strains were grown at 30°C in all experiments.

### Comparative genomics and phylogenetics

Histone H3 proteins were identified across eukaryotes using a taxonomically diverse dataset made up of genome- and transcriptome-predicted proteomes obtained from UniProt (*48*), EukProt V3 (*49*), and the SRA (Short Read Archive) (*n* = 184, downloaded 26 June 2024). To balance the dataset, the best two proteomes per genus based on BUSCO (Benchmarking Universal Single Copy Orthologs) (*50*) completeness were selected from UniProt. For animals, fungi, and plants, stricter taxonomic criteria were used to reduce overrepresentation, by selecting the best proteome per phyla for animals (*n* = 17), the best proteome per order for plants (max three per class, *n* = 6), and the best proteome per class for fungi (max two per phyla, *n* = 12). The dataset was also supplemented with transcriptome-predicted proteomes (*n* = 6) obtained from EukProt V3 and two species of CRuMs (*Rigifila ramosa*: SRR5997435; *Diphylleia rotans*: SRR5997435).

Histone H3 homologs were identified from the eukaryotic dataset using hidden Markov models (HMMs) and phylogenetically curated. The eukaryotic dataset was searched using a previously developed histone H3 profile HMM (*51*) using HMMER v3.4 (*E* < 10^−5^, domE < 10^−5^), and hits greater than 300 amino acids were excluded to avoid mispredicted proteins (*52*). To further exclude nonhomologous regions, the histone H3 HMM was mapped to each of the resulting proteins using HMMScan (*E* < 10^−5^, domE < 10^−5^) and corresponding regions were extracted. To remove redundancy resulting from recent paralogs, identical proteins derived the same species were clustered using CD-HIT v4.8.1 (*53*). To discriminate between core H3 and centromeric H3, the clustered proteins were aligned using the L-INS-i algorithm of MAFFT v7.5.20 and trimmed with a gap threshold of 30% using trimAl v1.4.rev15 (*54*, *55*). A phylogeny was then inferred using IQ-Tree v2.2.6 using the LG4M+R9 substitution model, as selected by ModelFinder based on the Bayesian information criterion (*56*, *57*). Topological support was assessed using Shimodaira-Hasegawa approximate likelihood ratio tests (*n* = 1000) (*58*). The phylogeny was visualized in FigTree v1.4 (http://tree.bio.ed.ac.uk/software/figtree/) and iTOL v6 (*59*). After isolating core H3 homologs, histones were realigned and classified as containing or lacking C110 based on the presence or absence of a cysteine within five aligned positions of C110, based on manual inspection of the alignment using AliView v1.28.

To examine histones across the ascomycetes, an additional database containing genome-predicted proteomes from UniProt was assembled (downloaded 23 November 2023). In particular, the best proteome per species based on BUSCO completeness was obtained for the Saccharomycotina (*n* = 80) and the best proteome per order for other Ascomycetes (*n* = 51) were collected. Histones, including those belonging to the H3, H4, H2A, and H2B families, were identified and extracted as described above. H3C110 distributions were visualized across the ascomycete phylogeny using IToL based on a species tree inferred using a set of 242 previously curated phylogenetic marker genes (*60*). Individual proteins were identified from reference proteomes using HMMER, and single gene phylogenies were generated with IQ-Tree (LG+G4 model, fast mode) and manually curated to exclude paralogs. Orthologs were realigned using L-INS-I and trimmed with a gap threshold of 80%, and a phylogeny was generated using IQ-Tree and the Q.yeast+I+R10 substitution model, selected using ModelFinder. Statistical support was inferred using ultrafast bootstraps (*n* = 1000) (*61*). Last, using the ascomycete histones, coevolving sites were identified using GroupSim and the Blosum62 matrix (*62*), amino acid context was assessed using WebLogo (*63*), and protein structures were visualized using PyMOL v2.5.0.

### Spot tests

Cells from exponentially growing cultures were 10-fold serially diluted and spotted on agar plates with various media conditions as indicated in the figures. Spotted plates were incubated at 30°C for up to 6 days and imaged daily. Images shown in the figures were captured when sufficient growth had occurred, and growth differences could be assessed (typically 3 to 5 days).

### Redox-sensitive GFP reporter plasmid

The gene encoding for roGFP2 was cloned from pRSETB-ro2GFP (Addgene, no. 82366) (*28*) and introduced into a modified pRS416 backbone containing TDH3 promoter and ADH1 terminator (URA3 PTDH3 ADH1term CEN6/ARSH4). The reduction-oxidation–sensitive GFP (roGFP) was created by substituting surface-exposed residues with cysteines on the *Aequorea victoria* GFP to allow disulfide bond formation under oxidizing conditions (*28*).

### Flow cytometry

Single colonies of cells bearing the Cup2 reporter plasmid or the roGFP2 reporter plasmid were grown in liquid SC-ura or SCEG-ura media overnight and diluted in fresh media the next day and allowed to grow to exponential phase before flow cytometry analysis. Replicate experiments were from different clones from the same transformation. Cells were directly assayed in the liquid media using a BD LSRFortessa X-20 instrument. Green fluorescence signal was collected using a 488-nm laser for excitation and a 515- to 545-nm band-pass filter for emission. For assaying roGFP2, the green fluorescence signal was excited with lasers simultaneously at 410 and 488 nm and emission for both excitation wavelengths was collected using 515- to 545-nm band-pass filter. For each replicate experiment, data from 30,000 events were collected and analyzed for each clone and the average profiles of four to eight independent transformants were reported.

### Inductively coupled plasma mass spectrometry

Cells from logarithmically growing cells were collected and washed twice in 1 mM EDTA for removal of extracellular metals and once in water. Cell pellets were frozen and stored at −20°C until further processed for ICP–tandem mass spectrometry (MS/MS. Cell pellets were overlaid with 70% nitric acid and digested at room temperature for 24 hours, followed by incubation at 65°C for at least 2 hours, before being diluted to a final nitric acid concentration of 2% (v/v). ICP-MS was performed on the Agilent 8900 ICP-MS/MS. ^63^Cu and ^56^Fe were used to determine the total cellular copper and iron levels using He as a cell gas and ^89^Y as an internal standard (Inorganic Ventures MSY-100PPM) and calibrated with an environmental calibration standard (Agilent, 5183-4688). All measurements were within the calibrated linear ranges. The average of four technical replicate measurements was used from five independent biological samples where the technical variation never exceeded 5% for any individual sample. ICP-MS/MS data were then analyzed using the Agilent ICP-MS MassHunter software (v4.4).

### RNA-seq and differential gene expression analysis

Cells grown in fermentative (SC) or oxidative media (SCEG) were collected by centrifugation and frozen at −20°C until processed for RNA extraction and RNA sequencing (RNA-seq). RNA was extracted using previously published methods (*4*) from four independent experiments. Contaminating DNA was digested using Turbo DNase (Thermo Fisher Scientific). Sequencing libraries were prepared and sequenced by the UCLA Technology Center for Genomics & Bioinformatics. High-throughput sequencing was performed on Illumina’s NovaSeq system. Total read count per library ranged from ~8 million to 15 million. Demultiplexed reads, in FASTQ file format, were aligned in a strand-specific manner to the R64-1-1 S288C reference genome assembly (sacCer3) using HISAT2. Assigning and counting reads for 6692 annotated open reading frames were performed using featureCounts. Determination of adjusted *P* values for differential gene expression comparisons was done using DESeq2.

### Mass spectrometry analysis of metabolites

Cells (6 × 10^8^) from exponentially growing cultures were collected by centrifugation. The cells were washed once in 43% ice-cold methanol-water solution. To extract the metabolites, the washed cells were resuspended in 200 μl of ice-cold (stored at −80°C) 80% methanol containing 10 nM trifluoromethanesulfonate (TFMS). The resuspended cells were vortexed for 5 s, frozen immediately in liquid nitrogen for 5 min, and then allowed to thaw in an ice bath for 5 min. The cells were pelleted at 16,000*g* to collect the supernatant. The cell pellet was resuspended in another 100 μl of methanol-TFMS solution and pelleted to collect the supernatant. The combined supernatant was dried under vacuum, and the dried metabolites were stored at −80°C until mass spectrometry analysis. Extracted dried metabolites were reconstituted in a 50% acetonitrile:50% dH_2_O solution and processed further for analysis by mass spectrometry as previously described (*7*).

### Yeast RLS assay

RLSs of yeast strains were determined by using a microfluid platform as previously described (*35*, *36*) and analyzed by an Invitrogen EVOS FL Auto Imaging System. Briefly, cells were grown overnight in filter-sterilized YPD or glycerol (YPGlycerol) medium, diluted 20-fold with fresh medium, and loaded onto a microfluidic chip. Medium flow speed was set at 1 μl/min, and pictures were taken at 15-min intervals for 65 hours. The microfluidic chips were maintained at 30°C for the duration of the experiment. The pictures were taken using an EVOS FL Auto system (Thermo Fisher Scientific) using a 20x objective. Time-lapse image series were analyzed using ImageJ (NIH). Statistical assessment of life-span differences was determined using the Wilcoxon rank sum test in the R statistics software package.
