# Supplementary material for: Histone H3 cysteine 110 enhances iron metabolism and modulates replicative life span in Saccharomyces cerevisiae
Source: Sci Adv. 2025 Apr 11;11(15):eadv4082. doi: 10.1126/sciadv.adv4082 (PMC11988410; doi:10.1126/sciadv.adv4082)
Supplement: Supplementary file 1 — Figs. S1 to S5 Table S1 Legend for data S1 [file sciadv.adv4082_sm.pdf]

Science Advances
AAAS

Supplementary Materials for

Histone H3 cysteine 110 enhances iron metabolism and modulates replicative life span in *Saccharomyces cerevisiae*

Chen Cheng *et al.*

Corresponding author: Siavash K. Kurdistani, skurdistani@mednet.ucla.edu;
Nicholas A. T. Irwin, nicholas.irwin@gmi.oeaw.ac.at

Sci. Adv. **11**, eadv4082 (2025)
DOI: 10.1126/sciadv.adv4082

The PDF file includes:

Figs. S1 to S5
Table S1
Legend for data S1

Other Supplementary Material for this manuscript includes the following:

Data S1

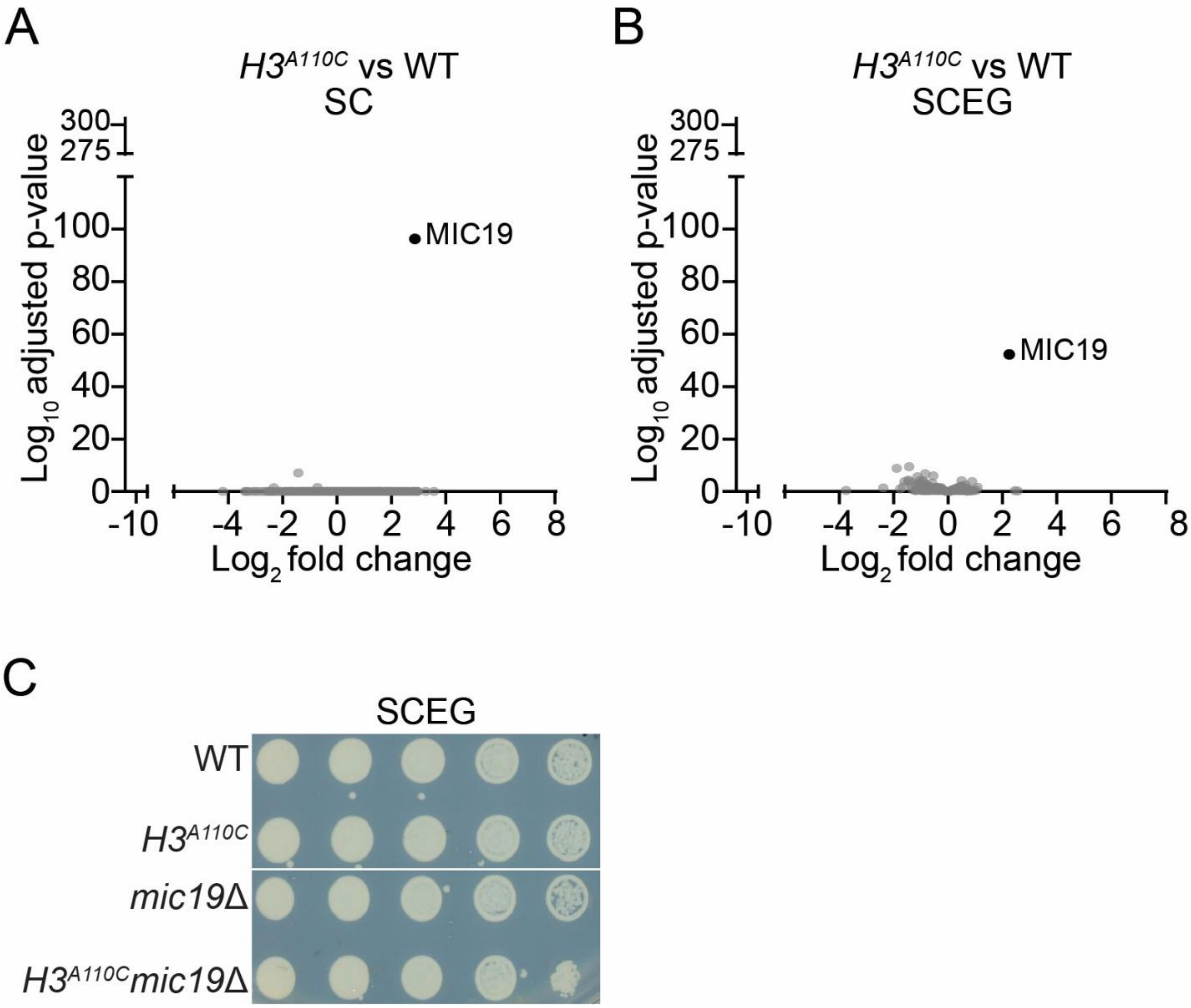

Figure S1. Reintroduction of H3C110 into *S. cerevisiae* does not substantially alter gene expression in otherwise wildtype background. Volcano scatter plots illustrating the relationship between changes in gene expression of the $H3^{A110C}$ vs WT strains grown in (**A**) fermentative (SC) or (**B**) oxidative (SCEG) medium and the corresponding level of significance. Notice that only the MIC19 gene is substantially and significantly upregulated under either growth condition. (**C**) Spot test assays of the indicated strains on oxidative medium (SCEG).

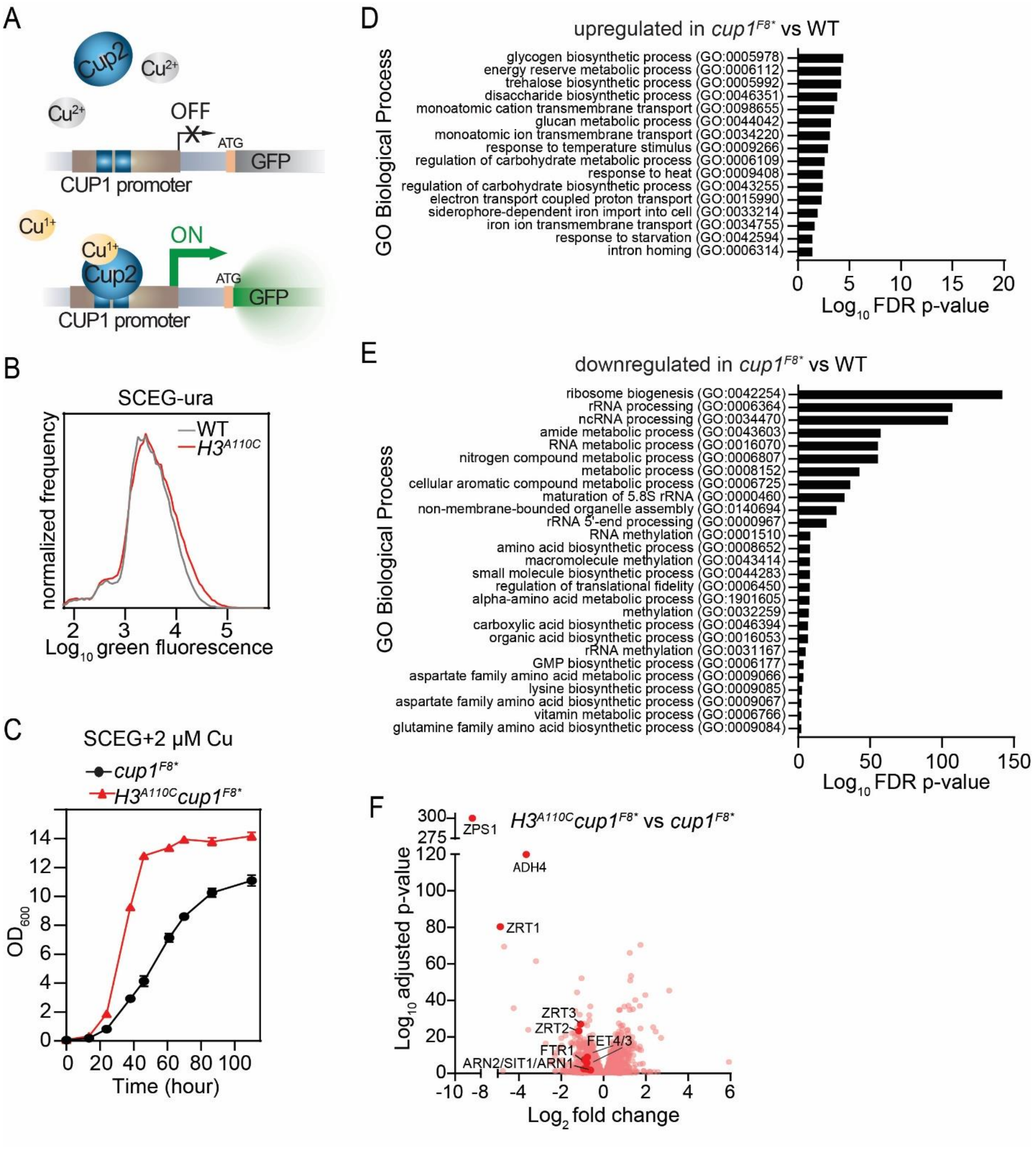

Figure S2. Reintroduction of H3C110 into *S. cerevisiae* increases intracellular Cu^{1+} levels. (**A**) Graphic representation of the reporter assay used to determine intracellular Cu^{1+}. (**B**) Average flow cytometry distributions of cells containing the p(CUP1)-GFP plasmid grown in oxidative medium (SCEG) from eight experiments. Baseline copper concentrations in SCEG is ~0.2 μM. (**C**) Growth curves of the indicated strains in oxidative medium (SCEG) with 2 μM additional copper in the form of $CuSO_4$. Baseline copper level in SCEG is 0.25 μM. (**D**) Significant gene ontologies among the genes upregulated in *cup1*^{F8*} strain vs WT are shown as

bar graph. (**E**) Same as D for the downregulated genes in *cup1*^{F8*} strain vs WT. (**F**) Volcano scatter plots illustrating the relationship between changes in gene expression of the indicated strains and the corresponding level of significance. Several genes involved in iron homeostasis are indicated.

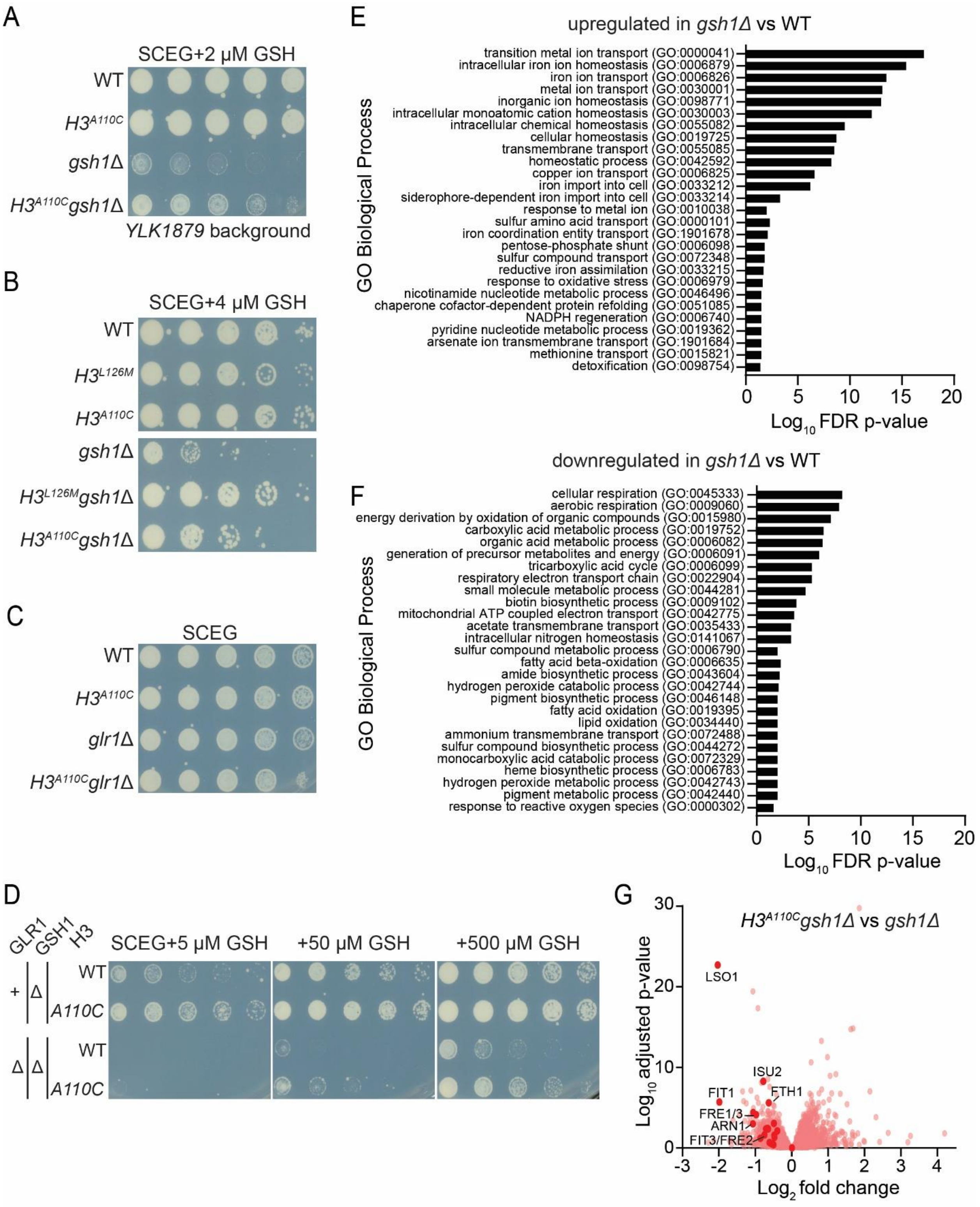
A
SCEG+2 μM GSH
WT
H3A110C
gsh1Δ
H3A110Cgsh1Δ
YLK1879 background
B
SCEG+4 μM GSH
WT
H3L126M
H3A110C
gsh1Δ
H3L126Mgsh1Δ
H3A110Cgsh1Δ
C
SCEG
WT
H3A110C
glr1Δ
H3A110Cglr1Δ
D
GLR1 GSH1 H3
SCEG+5 μM GSH
+50 μM GSH
+500 μM GSH
+ Δ WT
A110C
Δ Δ WT
A110C
E
upregulated in gsh1Δ vs WT
GO Biological Process
transition metal ion transport (GO:0000041)
intracellular iron ion homeostasis (GO:0006879)
iron ion transport (GO:0006826)
metal ion transport (GO:0030001)
inorganic ion homeostasis (GO:0098771)
intracellular monoatomic cation homeostasis (GO:0030003)
intracellular chemical homeostasis (GO:0055082)
cellular homeostasis (GO:0019725)
transmembrane transport (GO:0055085)
homeostatic process (GO:0042592)
copper ion transport (GO:0006825)
iron import into cell (GO:0033212)
siderophore-dependent iron import into cell (GO:0033214)
response to metal ion (GO:0010038)
sulfur amino acid transport (GO:0000101)
iron coordination entity transport (GO:1901678)
pentose-phosphate shunt (GO:0006098)
sulfur compound transport (GO:0072348)
reductive iron assimilation (GO:0033215)
response to oxidative stress (GO:0006979)
nicotinamide nucleotide metabolic process (GO:0046496)
chaperone cofactor-dependent protein refolding (GO:0051085)
NADPH regeneration (GO:0006740)
pyridine nucleotide metabolic process (GO:0019362)
arsenate ion transmembrane transport (GO:1901684)
methionine transport (GO:0015821)
detoxification (GO:0098754)
0 5 10 15 20
Log10 FDR p-value
F
downregulated in gsh1Δ vs WT
GO Biological Process
cellular respiration (GO:0045333)
aerobic respiration (GO:0009060)
energy derivation by oxidation of organic compounds (GO:0015980)
carboxylic acid metabolic process (GO:0019752)
organic acid metabolic process (GO:0006082)
generation of precursor metabolites and energy (GO:0006091)
tricarboxylic acid cycle (GO:0006099)
respiratory electron transport chain (GO:0022904)
small molecule metabolic process (GO:0044281)
biotin biosynthetic process (GO:0009102)
mitochondrial ATP coupled electron transport (GO:0042775)
acetate transmembrane transport (GO:0035433)
intracellular nitrogen homeostasis (GO:0141067)
sulfur compound metabolic process (GO:0006790)
fatty acid beta-oxidation (GO:0006635)
amide biosynthetic process (GO:0043604)
hydrogen peroxide catabolic process (GO:0042744)
pigment biosynthetic process (GO:0046148)
fatty acid oxidation (GO:0019395)
lipid oxidation (GO:0034440)
ammonium transmembrane transport (GO:0072488)
sulfur compound biosynthetic process (GO:0044272)
monocarboxylic acid catabolic process (GO:0072329)
heme biosynthetic process (GO:0006783)
hydrogen peroxide metabolic process (GO:0042743)
pigment metabolic process (GO:0042440)
response to reactive oxygen species (GO:0000302)
0 5 10 15 20
Log10 FDR p-value
G
H3A110Cgsh1Δ vs gsh1Δ
Log10 adjusted p-value
0 10 20 30
LSO1
ISU2
FIT1
FTH1
FRE1/3
ARN1
FIT3/FRE2
-3 -2 -1 0 1 2 3 4
Log2 fold change

Figure S3. Increasing the copper reductase activity histone H3 decreases the glutathione requirement in *S. cerevisiae*. (**A-B**) Spot test assays of the indicated strains on oxidative medium (SCEG) with glutathione (GSH) supplemented. Note that SCEG contains no glutathione. (**C**) Spot test assays of the indicated strains on oxidative medium (SCEG). Note that the *glr1Δ* strain does not require supplemental GSH for growth in oxidative media lacking glutathione. (**D**) Spot test assays of the indicated strains on oxidative medium (SCEG) supplemented with the indicated amounts of GSH. Note that SCEG contains no glutathione. (**E**) Significant gene ontologies among the genes upregulated in *gsh1Δ* strain vs WT are shown as bar graph. (**F**) Same as E for the downregulated genes in *gsh1Δ* strain vs WT. (**G**) Volcano scatter plots illustrating the relationship between changes in gene expression of the indicated strains and the corresponding level of significance. Several genes involved in iron homeostasis are indicated.

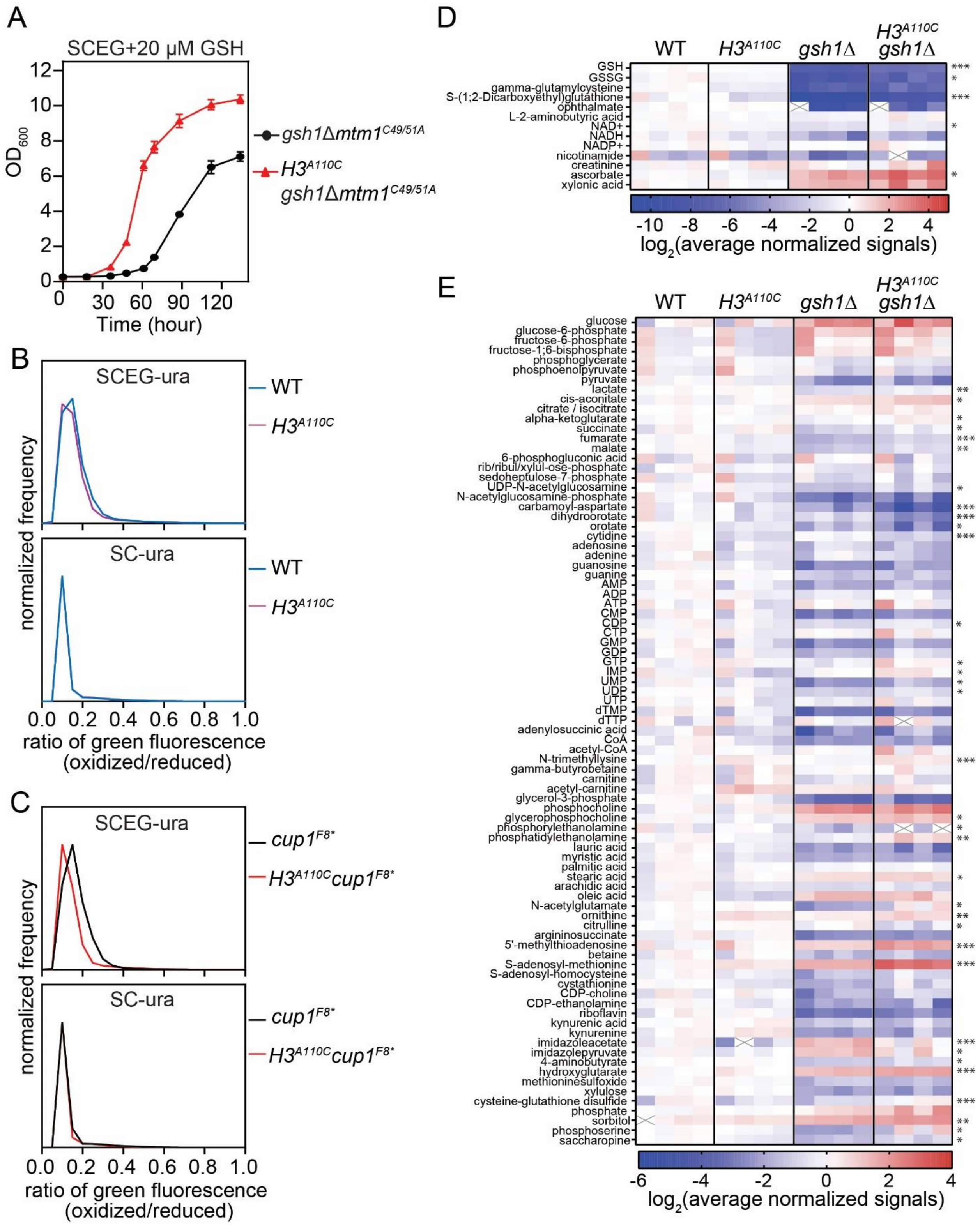

Figure S4. Reintroduction of H3C110 into *S. cerevisiae* improves cellular redox and levels of several metabolites. (**A**) Growth curves of the indicated strains in oxidative medium

supplemented with glutathione (GSH). Note that the cysteine mutations in Mtm1 likely diminish its function. **(B-C)** Average normalized flow cytometry distributions of cells containing the redox sensitive ro2GFP grown in the indicated media from eight experiments. The x-axis indicates the ratio of oxidized/reduced green fluorescence signal. (**D-E**) Heatmap showing intracellular levels of the indicated metabolites in the specified strains from four independent experiments. The scale represents log2 average-normalized levels of each metabolite. Missing datapoints are denoted by X. P values indicate the significance of comparisons between *gsh1Δ* and $H3^{A110C}$*gsh1Δ* datapoints (*p < 0.05, **p < 0.01; ***p < 0.001; t-test).

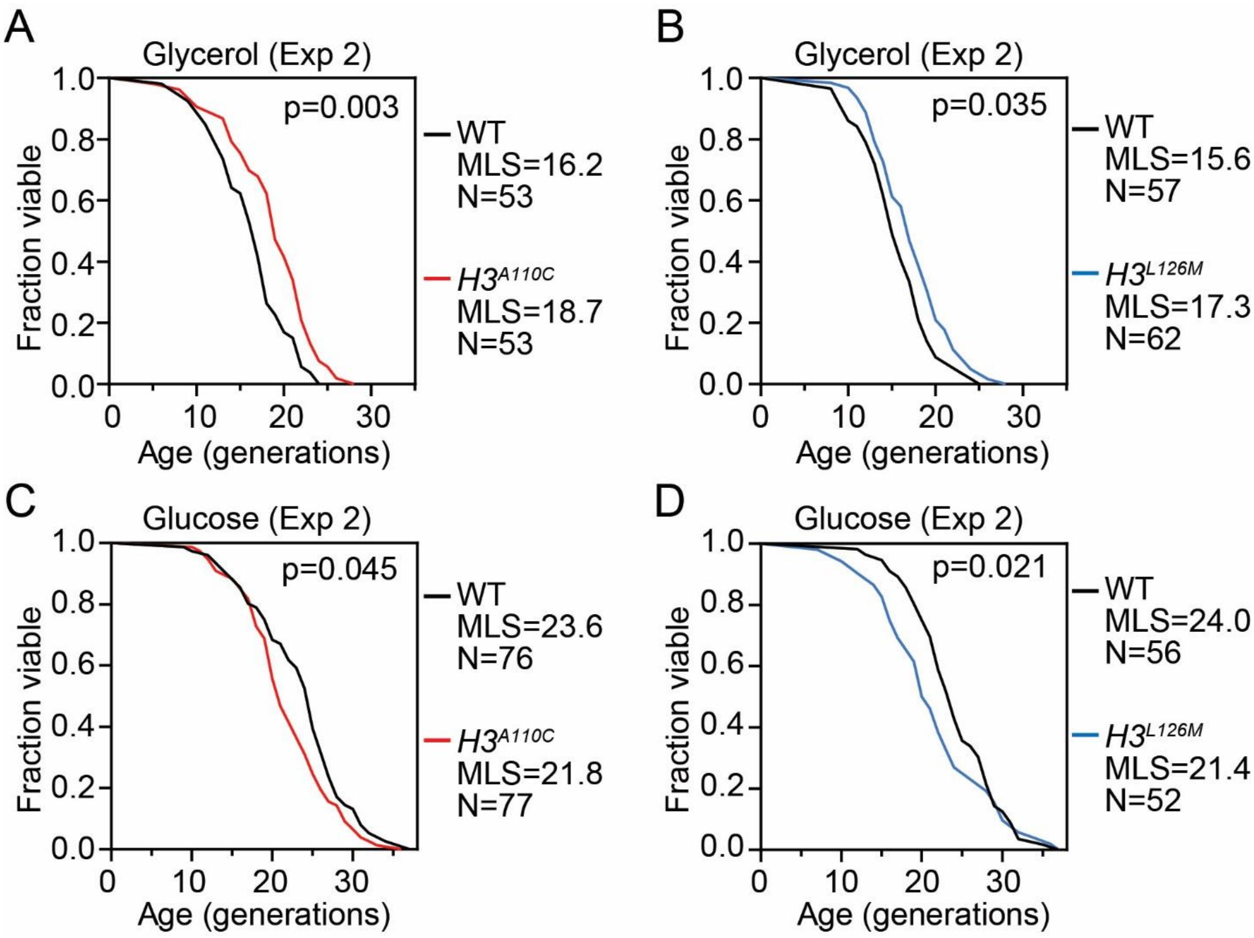

Figure S5. Gain-of-function mutations in the copper reductase activity of histone H3 extend replicative lifespan in oxidative medium but reduce it in fermentative medium. (**A-B**) Replicative lifespan analysis of strains with H3A110C or H3L126M mutation grown in oxidative medium (YPGlycerol) in an independent experiment relative to Fig. 5. Median lifespan (MLS) in days and number of cells analyzed are indicated. (**C-D**) Same as in A-B, but for cells cultured in fermentative medium (YPD). P values were calculated using the Wilcoxon rank-sum test.

Supplementary Table 1. A list of strains and their genotypes used in this study.

**Strain name**	**Strain short name**	**Genotype**	**Reference**
**BY4741**	parental strain	*MAT*a *his3Δ1 leu2Δ0 met15Δ0 ura3Δ0*	Attar et al., 2020
**OCY1131**	Wildtype (WT)	*MAT*a *his3Δ1 leu2Δ0 met15Δ0 ura3Δ0, (hht2Δ::ura3)Δ::HHT2*	Attar et al., 2020
**NAY616**	*$H3^{A110C}$*	*MATa his3Δ1 leu2Δ0 met15Δ0 ura3Δ0, (hht2Δ::ura3)Δ::HHT2, hht2-A110C, hht1-A110C*	Attar et al., 2020
**NTY062**	*$H3^{L126M}$*	*MATa his3Δ1 leu2Δ0 met15Δ0 ura3Δ0, (hht2Δ::ura3)Δ::HHT2, hht2-L126M, hht1-L126M*	Tod et al., 2024
**OCY1981**	*$cup1^{F8*}$*	*MATa his3Δ1 leu2Δ0 met15Δ0 ura3Δ0, (hht2Δ::ura3)Δ::HHT2, cup1-F8stop*	Attar et al., 2020
**OCY1984**	*$H3^{A110C}cup1^{F8*}$*	*MATa his3Δ1 leu2Δ0 met15Δ0 ura3Δ0, (hht2Δ::ura3)Δ::HHT2, hht2-A110C, hht1-A110C, cup1-F8stop*	Attar et al., 2020
**OCY2371**	*gsh1Δ*	*MATa his3Δ1 leu2Δ0 met15Δ0 ura3Δ0, (hht2Δ::ura3)Δ::HHT2, gsh1Δ::KanMX6*	Attar et al., 2020
**OCY2373**	*$H3^{A110C}$gsh1Δ*	*MATa his3Δ1 leu2Δ0 met15Δ0 ura3Δ0, (hht2Δ::ura3)Δ::hht2-A110C hht1-A110C, gsh1Δ::KanMX6*	Attar et al., 2020
**CCY0241**	*$H3^{L126M}$gsh1Δ*	*MATa his3Δ1 leu2Δ0 met15Δ0 ura3Δ0, (hht2Δ::ura3)Δ::HHT2, hht2-L126M, hht1-L126M, gsh1Δ::KanMX6*	This study
**CCY0211**	*Fet3Δ*	*MATa his3Δ1 leu2Δ0 met15Δ0 ura3Δ0, (hht2Δ::ura3)Δ::HHT2, fet3Δ::KanMX6*	This study
**CCY0212**	*$H3^{A110C}$fet3Δ*	*MATa his3Δ1 leu2Δ0 met15Δ0 ura3Δ0, (hht2Δ::ura3)Δ::hht2-A110C hht1-A110C, fet3Δ::KanMX6*	This study
**CCY0213**	*gsh1Δfet3Δ*	*MATa his3Δ1 leu2Δ0 met15Δ0 ura3Δ0, (hht2Δ::ura3)Δ::HHT2, gsh1Δ::KanMX6, fet3Δ::HphMX4*	This study
**CCY0214**	*$H3^{A110C}$gsh1Δfet3Δ*	*MATa his3Δ1 leu2Δ0 met15Δ0 ura3Δ0, (hht2Δ::ura3)Δ::hht2-A110C hht1-A110C, gsh1Δ::KanMX6, fet3Δ::HphMX4*	This study
**CCY0465**	*gsh1Δ$mtm1^{C49/51A}$*	*MATa his3Δ1 leu2Δ0 met15Δ0 ura3Δ0, (hht2Δ::ura3)Δ::HHT2, gsh1Δ::KanMX6, $mtm1^{C49/51A}$*	This study
**CCY0466**	*$H3^{A110C}$gsh1Δ$mtm1^{C49/51A}$*	*MATa his3Δ1 leu2Δ0 met15Δ0 ura3Δ0, (hht2Δ::ura3)Δ::hht2-A110C hht1-A110C, gsh1Δ::KanMX6, $mtm1^{C49/51A}$*	This study
**CCY0141**	*glr1Δ*	*MATa his3Δ1 leu2Δ0 met15Δ0 ura3Δ0, (hht2Δ::ura3)Δ::HHT2, glr1Δ*	This study
**CCY0142**	*$H3^{A110C}$glr1Δ*	*MATa his3Δ1 leu2Δ0 met15Δ0 ura3Δ0, (hht2Δ::ura3)Δ::hht2-A110C hht1-A110C, glr1Δ*	This study
**CCY0143**	*gsh1Δglr1Δ*	*MATa his3Δ1 leu2Δ0 met15Δ0 ura3Δ0, (hht2Δ::ura3)Δ::HHT2, gsh1Δ::KanMX6, glr1Δ*	This study
**CCY0144**	*$H3^{A110C}$gsh1Δglr1Δ*	*MATa his3Δ1 leu2Δ0 met15Δ0 ura3Δ0, (hht2Δ::ura3)Δ::hht2-A110C hht1-A110C, gsh1Δ::KanMX6, glr1Δ*	This study
**OCY2131**	*cox17Δ*	*MATa his3Δ1 leu2Δ0 met15Δ0 ura3Δ0, (hht2Δ::ura3)Δ::HHT2, cox17Δ::KanMX6*	This study
**OCY2133**	*$H3^{A110C}$cox17Δ*	*MATa his3Δ1 leu2Δ0 met15Δ0 ura3Δ0, (hht2Δ::ura3)Δ::hht2-A110C hht1-A110C, cox17Δ::KanMX6*	This study
**CCY0361**	*mic19Δ*	*MATa his3Δ1 leu2Δ0 met15Δ0 ura3Δ0, (hht2Δ::ura3)Δ::HHT2, mic19Δ::HphMX4*	This study
**CCY0362**	*$H3^{A110C}$mic19Δ*	*MATa his3Δ1 leu2Δ0 met15Δ0 ura3Δ0, (hht2Δ::ura3)Δ::hht2-A110C hht1-A110C, mic19Δ::HphMX4*	This study
**YLK1879**	YLK WT	*MAT*a	Attar et al., 2020
**OCY3005**	YLK *$H3^{A110C}$*	*MAT*a *hht2-A110C, hht1-A110C*	This study
**CCY1121**	YLK *gsh1Δ*	*MAT*a, *gsh1Δ::KanMX6*	This study
**CCY1122**	YLK *$H3^{A110C}$gsh1Δ*	*MAT*a *hht2-A110C, hht1-A110C, gsh1Δ::KanMX6*	This study

Supplementary Excel File. The file contains raw and normalized metabolomics data as indicated in the “Note” tab.
